# Insights into the possible role of IFNG and IFNGR1 in Kala-azar and Post Kala-azar Dermal Leishmaniasis in Sudanese patients

**DOI:** 10.1186/s12879-014-0662-5

**Published:** 2014-12-03

**Authors:** Mohamed A M Salih, Michaela Fakiola, Mohamed H Abdelraheem, Brima M Younis, Ahmed M Musa, Ahmed M ElHassan, Jenefer M Blackwell, Muntaser E Ibrahim, Hiba S Mohamed

**Affiliations:** Institute of Endemic Disease, University of Khartoum, Khartoum, Sudan; Central laboratory, Ministry of Science and Technology, Khartoum, Sudan; Department of Medicine and Department of Pathology, Cambridge Institute for Medical Research, University of Cambridge, Cambridge, UK; Telethon Kids Institute, The University of Western Australia, Crawley, Australia

**Keywords:** PKDL, Visceral leishmaniasis, Polymorphisms, RNA expression, IFNG, IFNGR1, Rare variants, Sudan

## Abstract

**Background:**

Little is known about the parasite/host factors that lead to Post Kala-azar Dermal Leishmaniasis (PKDL) in some visceral leishmaniasis (VL) patients after drug-cure. Studies in Sudan provide evidence for association between polymorphisms in the gene (*IFNGR1*) encoding the alpha chain of interferon-γ receptor type I and risk of PKDL. This study aimed to identify putative functional polymorphisms in the *IFNGR1* gene, and to determine whether differences in expression of interferon-γ (IFNG) and IFNGR1 at the RNA level are associated with pathogenesis of VL and/or PKDL in Sudan.

**Methods:**

Sanger sequencing was used to re-sequence 841 bp of upstream, exon1 and intron1 of the *IFNGR1* gene in DNA from 30 PKDL patients. LAGAN and SYNPLOT bioinformatics tools were used to compare human, chimpanzee and dog sequences to identify conserved noncoding sequences carrying putative regulatory elements. The relative expression of IFNG and IFNGR1 in paired pre- and post-treatment RNA samples from the lymph nodes of 24 VL patients, and in RNA samples from skin biopsies of 19 PKDL patients, was measured using real time PCR. Pre- versus post-treatment expression was evaluated statistically using the nonparametric Wilcoxon matched pairs signed-rank test.

**Results:**

Ten variants were identified in the 841 bp of sequence, four of which are novel polymorphisms at -77A/G, +10 C/T, +18C/T and +91G/T relative to the *IFNGR1* initiation site. A cluster of conserved non-coding sequences with putative regulatory variants was identified in the distal promoter of *IFNGR1*. Variable expression of IFNG was detected in lymph node aspirates of VL patients before treatment, with a marked reduction (*P* = 0.006) in expression following treatment. IFNGR1 expression was also variable in lymph node aspirates from VL patients, with no significant reduction in expression with treatment. IFNG expression was undetectable in the skin biopsies of PKDL cases, while IFNGR1 expression was also uniformly low.

**Conclusions:**

Uniformly low expression of IFN and IFNGR1 in PKDL skin biopsies could explain parasite persistence and is consistent with prior demonstration of genetic association with *IFNGR1* polymorphisms. Identification of novel potentially functional rare variants at *IFNGR1* makes an important general contribution to knowledge of rare variants of potential relevance in this Sudanese population.

**Electronic supplementary material:**

The online version of this article (doi:10.1186/s12879-014-0662-5) contains supplementary material, which is available to authorized users.

## Background

Post Kala-azar Dermal Leishmaniasis (PKDL) is a complication of treatment for patients with visceral leishmaniasis (VL) caused by *Leishmania donovani* and results in the presence of multiple hypopigmented papules or nodules on the skin. PKDL patients are considered a potential reservoir for anthroponotic leishmaniasis owing to the high number of parasites in their skin [1],[2]. Taking into consideration the high incidence of PKDL cases in East Africa, and the ability to experimentally infect *P. orientalis* fed on VL patients, Elnaiem and colleagues [3] concluded that transmission from human to human is common. Although it has been suggested that immune suppression may occur in PKDL patients, allowing renewed multiplication of latent parasites from the viscera or re-infection of the skin, the pathogenesis of disease remains unclear [4]. According to studies conducted in Sudan, reactivation of leishmania-specific lymphocyte is considered to be the major underlying mechanism in PKDL development [5]. Inadequate treatment, host genetics, and immune response may all play a role in the development of PKDL.

Susceptibility or resistance to leishmaniasis shows marked variation within and between genetically diverse human populations. Genetic analyses of VL in the Sudanese population revealed that PKDL induced by *L. donovani* infection is influenced by polymorphisms in the gene, *IFNGR1*, encoding the receptor α-chain for interferon-γ [6],[7]. Failure of macrophages to become activated to kill parasites during PKDL may be due to low expression of IFNGR1 which may lead to a gap in downstream signalling [8],[9].

Transcriptional regulation is considered to be an important step in gene regulation because it controls the number of copies of primary transcripts, which is the first type of biomolecule produced during the gene expression process. Several *IFNGR1* polymorphisms/mutations were identified in patients with different diseases. Sequence analysis of 2.5 kb spanning the promoter region to exon1 of the *IFNGR1* gene in 24 Japanese individuals, 12 unaffected and 12 atopic dermatitis patients, identified nine polymorphisms, five of which were detected in the promoter region while the other four variants were found in exon 1 [10]. The -56C/T promoter polymorphism, where the T allele was associated with higher *IFNGR1* transcriptional activity, represented a genetic risk factor for ocular complications of atopic dermatitis [10]. Koch and co-workers [11] sequenced 1400 bp upstream of the transcriptional start site in 34 randomly selected Gambian umbilical cord blood samples and 36 Gambian tuberculosis (TB) patients. They identified four polymorphisms, -470delTT, -270 T/C, -56C/T, and +95 T/C. Genotyping these polymorphisms in cord blood controls and severe malaria patients showed that individuals carrying the double deletion at -470delTT were protected against severe malaria in the Mandinka ethnic group, while heterozygosity at the -56C/T SNP was specifically associated with protection from cerebral malaria [11]. Awomoyi and co-workers [12] sequenced a further 32 patients with pulmonary TB from The Gambia, but reported no further novel variants and found no disease associations for TB when comparing 320 smear positive TB cases with 320 matched controls. Polymorphism at the -56 T/C SNP is associated with the clinical outcome of HBV infection in Chinese adults, with the C allele associated with viral clearance and the T allele associated with viral persistence [13].

In Sudan, we found that risk of PKDL was associated with the T allele at the -270 T/C polymorphism, but not with the three polymorphisms -470delTT, -56C/T, or +95 T/C individually [7]. Nevertheless, global association with haplotypes comprising all four markers at *IFNGR1* was observed, associated with a significant bias in transmission of the (-470 bp_-270 bp_-56 bp_ + 95 bp) haplotype insTT_T_T_T and less than expected transmission of insTT_C_C_C. When compared with data on malaria associations from The Gambia, the results suggested a complex pattern of haplotypic variation at the *IFNGR1* promoter locus associated with different infectious disease in African populations, prompting us to explore further the possibility of additional promoter region polymorphisms in the Sudanese population. This study was therefore carried out to determine whether novel and putative functional variants occur in regulatory regions of the *IFNGR1* locus in Sudanese individuals, and to determine whether differences in expression of IFNG and IFNGR1 at the RNA level are associated with pathogenesis of VL and/or PKDL in an endemic region of Sudan.

## Methods

### Study subjects

Lymph node aspirates from 24 paired pre- and post-treatment (sodium stibogluconate, 20 mg/kg/day for 30 days) VL patients (14 males, 10 females; age range 5 to 30 years), and 19 skin biopsies from PKDL patients (12 males, 7 females; age range 5 to 30 years), were collected in 5× RNA later (AMBioN Inc., Austin, texas, USA) and kept at −20*°*C until RNA was isolated. Normal skin tissue RNA (STRATAGENE, Foster City CA, USA) was included as control. Peripheral blood mononuclear cells (PBMCs) from blood samples of an additional 30 PKDL patients (19 males, 11 females; age range 5 to 30 years) were collected in transport buffer and stored at 4°C until DNA was extracted. The patients were recruited from Kassab Hospital–Elgadarif state, Sudan, during 2008 and 2009. Fever, hepatomegaly, splenomegaly, anaemia, leukopenia and hypo-albuminemia were observed in most VL patients. Diagnosis was carried out microscopically by demonstration of *Leishmania* amastigotes from either lymph node or bone marrow aspirates.

### Ethical considerations

The study was approved by the Institutional Review Board known as the Ethics Committee of Institute of Endemic Diseases, University of Khartoum. Written informed consent was obtained from all adult participants, and from the parent or guardian where participants were less than 18 years old. All clinical investigations were conducted according to the principles expressed in the Declaration of Helsinki (http://www.wma.net/en/30publications/10policies/b3/index.html).

### Sequencing of *IFNGR1*

Genomic DNA was extracted from PBMCs using DNeasy Blood & Tissue Kit in accordance with the manufacturer’s instruction (QIAGEN). The 1029 bp genomic region of the *IFNGR1* gene targeted for sequencing included 841 bp of upstream sequence, plus exon1 and intron1 (Ensembl v73; http://browser.1000genomes.org). This target region was amplified from 30 PKDL DNA samples using primers: 5′AAACAGTAGGGCGGGGTAAG3′ and 5′AAATCAAATCGGCTTGACCA3′ designed using primer 3 software (http://frodo.wi.mit.edu/primer3/). Sanger sequencing reactions were outsourced to the Macrogen Company, Seoul, South Korea.

### Real time PCR

Total RNA was isolated from paired lymph node samples for the 24 pre- and post-treatment VL patients, and from skin biopsies for the PKDL patients using the RNeasy Micro kit (Qiagen, GmbH, Hilden, Germany) according to the manufacturer’s instruction. Sample quality and integrity was assessed by NanoDrop spectrophotometer (ND-1000). Complementary DNA (cDNA) was synthesized using MMLV reverse transcriptase kit (Stratagene) in accordance with the manufacturer’s instruction. The SYBER Green gene expression assay (2× Brilliant SYBER Green QPCR master mixes, STRATAGENE) was used to measure expression (MX3000P Real Time PCR system, STRATAGENE, Foster City CA, USA) of IFNG and IFNGR1, with β-Actin used as an endogenous control. Sense and antisense primers for IFNG, IFNGR1 and β-Actin were designed using primer 3 software (http://frodo.wi.mit.edu/primer3/) (Table 1). Controls without template were included in each plate. All samples were run in duplicate. Expression of target genes was normalized to endogenous control using the standard curve method for relative quantitation according to Stratagene’s instructions. Final results for PKDL samples are shown relative to the control skin tissue RNA. Results were analysed in GraphPad Prism (version 5.00 for Windows, Graph Pad Software, San Diego California USA, http://www.graphpad.com). Statistical differences between VL pre- and post-treatment groups were determined using the nonparametric Wilcoxon matched pairs signed-rank test.

**Table 1:** **Sense and anti-sense sequences for the housekeeping gene and cytokines**

Gene	Primer sequence	Product size (bp)
*B-Actin*	Fwd. 5′CTGTGGCATCCACGAAACTA 3′	200
	Rev. 5′AGTACTTGCGCTCAGGAGGA 3′	
*IFNG*	Fwd. 5′GTTTTGGGTTCTCTTGGCTGTTA 3′	113
	Rev. 5′AAAAGAGTTCCATTATCCGCTACATC 3′	
*IFNGR1*	Fwd. 5′GCCACAGGTCCCTGTTTTTA 3′	163
	Rev. 5′TCCAACCCTGGCTTTAACTC 3′	

### Bioinformatics analysis

Polymorphisms within the 841 bp fragment amplified upstream and across exon1 and intron1 of *IFNGR1* were identified using the BioEdit program (http://www.mbio.ncsu.edu/BioEdit/bioedit.html) [14]. Linkage disequilibrium between polymorphic variants was determined using Haploview v4.2 [15] (http://sourceforge.net/projects/haploview/). To identify regulatory polymorphisms within conserved non coding sequences (CNSs) upstream of the *IFNGR1* gene, a 3.5 kb region of genomic sequences and associated gene annotations for human, chimpanzee, and dog were exported from Ensembl (National Center for Biotechnology Information build 36; Ensembl release 73) in FASTA and General Feature File (GFF) formats, respectively. Global alignment of genomic sequences was performed in Multi-LAGAN (http://lagan.stanford.edu/lagan_web/index.shtml) [16],[17]. The annotated alignment was visualized in SynPlot (http://hscl.cimr.cam.ac.uk/syn_plot.html), and CNSs of a specified percentage were identified in SynPlot-Peaks (http://hscl.cimr.cam.ac.uk/syn_plot_peaks.html) [18]. To search for putative transcription factor binding sites (TFBSs) at SNP locations, the online software tools AliBaba [19] (version 2.1; http://www.gene-regulation.com/pub/programs.html) and MatInspector [20] ((Genomatix) Matrix Family Library Version 9.1) were used.

## Results

### Sequence analysis of *IFNGR1*

Associations between PKDL and functional polymorphisms in the *IFNGR1* promoter region [7] encouraged us to screen for additional SNPs or other variants within potential regulatory regions of the *IFNGR1*gene. Sequencing of 841 bp upstream, exon1 and intron1 of *IFNGR1* in 30 DNA from PKDL patients revealed ten genetic variants (Table 2). Four of these were not previously reported in public domain DNA databases (Ensembl v73; http://browser.1000genomes.org): *IFNGR1* -77A/G in the 5‴ promoter region, *IFNGR1* + 10C/T and *IFNGR1* + 18C/T in exon1, and *IFNGR1* + 91G/T in intron1. The +10 C/T polymorphism in exon1 of *IFNGR1* resulted in an amino acid change from Leucine (L) to Phenylalanine (F), which lies in the same class (Non Polar). No change in amino acid was associated with the +18C/T polymorphism. Substitution from A to G at the *IFNGR1* -77 bp polymorphism resulted in loss of the transcription factor binding sites for cAMP response element-binding (CREB) and hypoxia response element (HRE). Only the +91G/T polymorphism has a minor allele frequency >0.1 (Table 2). As such, it is the only novel polymorphism that could provide sufficient power to contribute to risk versus protective haplotypes that might account for the association with PKDL in this population. However, due to the low minor allele frequencies, strong linkage disequilibrium was only detected between the known SNPs *IFNGR1* + 95 T/C and *IFNGR1* -56 T/C (*r*^*2*^ = 0.71), in broad agreement with linkage disequilibrium between these two SNPs in the Yoruba (YRI) (*r*^*2*^ = 1), European (CEU) (*r*^*2*^ = 1), Japanese (JPN) (*r*^*2*^ = 1), and Han Chinese in Beijing (HCB) (*r*^*2*^ = 1) HapMap populations. Of interest, the novel SNP at +91 bp was not in strong linkage disequilibrium with the known SNPs as -56 bp (*r*^2^ = 0.01) or at +95 bp (*r*^2^ = 0.08). Therefore we conclude that it does not contribute to the association with PKDL previously observed for haplotypes involving these two markers [7].

**Table 2:** **List of IFNGR1 SNPS detected in Sudanese PKDL patients**

SNP	Region	Alleles	Position	Consequence (Loss or gain TFBS ^a^, AA change, splicing site)	MAF ^b^
rs1327474	5′-Promoter	A/G	−611	HOXC13, *GATA*	0.057
rs41401746	5′-Promoter	TT/--	−470	PU.1, Lyf-1	0.020
rs14183614	5′-Promoter	T/C	−270	-	0.017
rs7753590	5′-Promoter	T/G	−187	*SP1, EKLF*	0.035
**Novel**	**5′-Promoter**	**A/G**	**−77**	**CREB,** ***HRE***	**0.013**
rs2234711	5′-Promoter	T/C	−56	*MIZ1*	0.476
**Novel**	**Exon 1**	**C/T**	**+10**	**Leu/Phe**	**0.012**
**Novel**	**Exon 1**	**C/T**	**+18**	**Synonymous**	**0.023**
**Novel**	**Intron 1**	**G/T**	**+91**	**Splicing site**	**0.108**
rs7749390	Intron 1	T/C	+95	Splicing site	0.388

^a^TFBS, transcription factor binding site; grey shading, loss of TFBS; italics, gain of TFBS; AA = Amino Acid; ^b^MAF = minor allele frequency. Bold indicates novel variants found in this study.

### Bioinformatics analysis for conserved non-coding sequences (CNSs)

**Figure 1:**
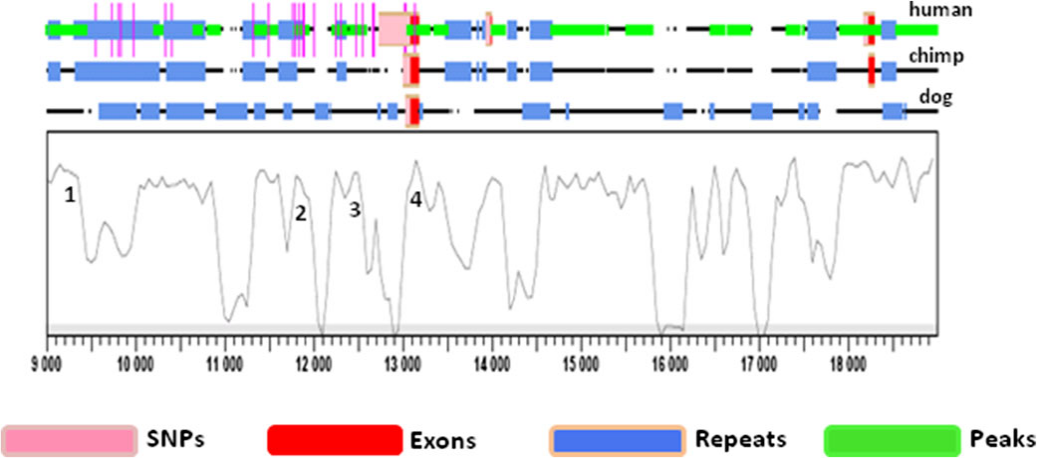
**Graphical representation of the** ***IFNGR1*** **upstream multiple alignment generated in SynPlot.** The sequences of each species are shown as lines interrupted by spaces corresponding to the gaps inserted for optimum global alignment. The horizontal axis represents the distance from the start of the alignment, and the vertical axis represents the percentage identity score generated by SynPlot (scale, 0%–100% sequence identity across all species). Peak regions that correspond to conserved noncoding sequences, as opposed to coding sequences or repeat features, are numbered. Other features are colour coded according to the key.

Sequencing of the immediate promoter region identified 4 novel variants in the Sudanese population. However, minor allele frequencies were low making it unlikely that these variants contributed on their own to risk and protective haplotypes for PKDL. Therefore *in silico* methods were employed to interrogate an extended region up to 3.5 kb upstream of the initiation start site of the *IFNGR1* gene. The strategy was to: (i) identify regions of conserved sequence (CNS) that are more likely to carry regulatory elements; (ii) identify TFBS within these CNS using predictive software tools; and (iii) determine whether known SNPs located within CNS cause loss or gain of relevant transcription factor binding activity. To identify CNSs, comparative sequence analysis was performed between human, chimpanzee, and dog for ~3.5 kb upstream region of *IFNGR1* gene. A number of CNSs were identified across the region based on the criteria of 70% similarity over at least 100 bp of ungapped alignment [21],[22]. Within this region we identified 3 CNSs in the intergenic region upstream of the *IFNGR1* gene (Figure 1) which we interrogated for public domain SNP information. In total we found information on 21 SNPs across the 3.5 kb regions upstream of the IFNGR1 initiation site, 13 of which fell within CNSs as annotated on Figure 1. No known SNPs were located within peak 1. Seven SNPs were identified in peaks 2 and 3, as presented in Table 3. Two out of the ten SNPs that were detected in the sequence analysis (Table 2) are located within the CNS labelled peak 4 which encompasses the 5′UTR (Figure 1). *IFNGR1* rs41401746 (−470 bp TT/-- INDEL) upstream of *IFNGR1* is positioned within peak 4, which showed 81% identity over 182 nucleotides across human, chimpanzee, and dog sequences. The rs141836145 (−270 bp T/C) SNP, which was found to be associated with PKDL in our previous study [7], is also located within peak 4. Four known SNPs (rs55961762, rs55640745, rs56300633, and rs17181653), also located within peak 4, were not polymorphic in the 30 PKDL samples that were sequenced (data not shown).

**Table 3:** **Known SNPs within CNS peaks identified in the region from the initiation site to 3.5 kb upstream of** ***IFNGR1***

SNP	Physical position bp	CNS peak ^a^	Alleles	MAF ^b^	Loss/gain TFBS ^c^
rs55995532	137541178	3	A/C	----	C/EBbeta
rs17175064	137541195	3	C/A	0.006	-
rs1327473	137541230	3	A/T	0.017	AP-1, C/EBPa1P
rs56273545	137541233	3	A/G	----	-
rs56178591	137541279	3	C/T	----	MAZ, ZNF263
rs17175057	137541297	3	G/A	EUR, 0.023	SP1, KLF7
rs17175050	137541521	2	A/T	0.009	*NF-1/GATA-1* ZBTB3

^a^Peaks are numbered in Figure 1. ^b^global MAF from SNPDB, −--- indicates no population data available in SNPDB, EUR indicates frequency in PGA European Panel. MAF, minor allele frequency; ^c^TFBS, transcription factor binding site; grey shading, loss of TFBS; italics, gain of TFBS.

Seven known SNPs in the SNPDB database fall within CNS peaks 2 and 3 located within the region 1000-2800 bp region upstream of *IFNGR1* (Table 3). Six of them, rs55995532, rs17175064, rs1327473, rs56273545, rs56178591, and rs17175057, are located within peak 3 which shows 79% identity across species over 154 nucleotides of alignment. The seventh SNP, rs17175050, is located within peak 2 which shows 81% identity across species over 365 nucleotides of alignment. Carriage of alternative alleles at rs55995532, rs1327473, and rs17175050, rs56178591, rs17175057 result in loss or gain of transcription factor binding sites for C/EBPbeta, AP-1, NF-1/GATA-1/ ZBTB3, MAZ/ ZNF263, and SP1/ KLF7, respectively (Table 3). However, public domain minor allele frequency information (Table 3) indicates that, as for the novel SNPs identified in the re-sequencing analysis, these SNPs are either known to be rare variants or likely to be in the situation where no allele frequency data is available in SNPDB.

### Expression of IFNG and IFNGR1 at the RNA level

**Figure 2:**
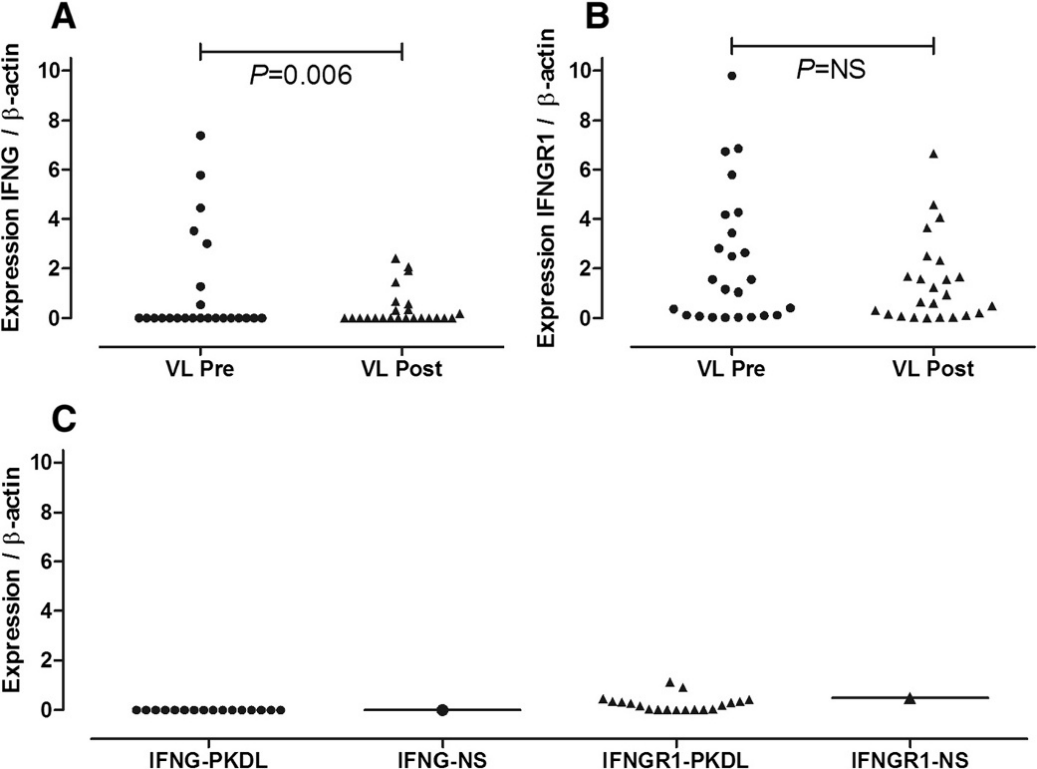
**Cytokine expression relative to the endogenous housekeeping gene β-actin in RNA from lymph node aspirates and skin biopsies from VL and PKDL patients. (A)** and **(B)** show IFNG and IFNGR1 expression, respectively, in paired lymph node aspirates taken pre- and post-treatment from 24 VL cases. P values for comparison of pre- and post-treatment samples are based on nonparametric Wilcoxon matched pairs signed-rank test. **(C)** shows IFNG and IFNGR1 expression in skin biopsies from 19 PKDL patients. Levels of the two cytokines relative to β-actin in commercially acquired normal skin (IFNG-NS and IFNGR1-NS) are shown by the horizontal bars.

To understand more about the possible role of *IFNG* and *IFNGR1* genes in the development of PKDL, their expression relative to an endogenous house-keeping gene was measured in RNA samples prepared from paired pre- and post-treatment lymph node aspirates from VL patients, and from skin biopsies from PKDL patients. Variable expression of IFNG was detected in lymph node aspirates of VL patients before treatment (Figure 2A), with a marked reduction (*P* = 0.006) in expression following treatment in paired pre- versus post-treatment samples. IFNGR1 expression was also variable in lymph node aspirates from VL patients, with no significant change in expression with treatment measured in paired pre- versus post-treatment samples. IFNG expression was undetectable in the skin biopsies of PKDL cases (Figure 2B), as it was in the normal commercial control skin. IFNGR1 expression was detectable but also uniformly low, especially relative to levels observed in the lymph nodes of VL patients.

## Discussion

*IFNGR1* gene polymorphisms were shown to be linked and associated specifically with PKDL in Sudan [6],[7]. To determine whether additional polymorphisms influence the development of this neglected disease, sequence analysis was performed across putative regulatory regions of the *IFNGR1* gene in 30 Sudanese PKDL patients. Four novel SNPs (*IFNGR1* -77A/G, *IFNGR1* + 10C/T, *IFNGR1* + 18C/T, and *IFNGR1* + 91G/T) located in the upstream region, exon1, and intron1 of the *IFNGR1* gene were identified. The SNP at +91G/T, along with the previously identified SNP +95 T/C, are located in/near the splicing site of the *IFNGR1* gene at the intron 1/exon 1 boundary [23]. Alternative alleles at these sites may decrease recognition of the adjacent exon, consequently preventing splicing out of this intron and altering the protein sequence and function. The A to G substitution at nucleotide -77 bp was also shown to result in loss or gain of the binding site for the transcription factors CREB and HRE, respectively. CREB plays a role in cell proliferation, differentiation, and survival. Once serine 133 of the CREB protein is phosphorylated. CREB interacts with its co-activator protein, CREB-binding protein (CBP), to initiate transcription of CREB-responsive genes [24]-[26]. A change from A to G at this position may disrupt this potential binding site, and thus affect the expression of IFNGR1. The gain of an HRE might also have important functional consequences. HREs are hypoxia response elements. Hypoxia promotes blood vessel formation, and in wounds it promotes the migration of keratinocytes and restoration of the epithelium [27]. However, none of the novel variants identified in our study occurred at a frequency in PKDL patients likely to contribute to the previously observed genetic associations, and the +91G/T was not in linkage disequilibrium with markers previously shown to be on risk and protective haplotypes. Therefore, whilst of interest as potential functional rare variants in this Sudanese population in general, the particular SNPs identified here were unlikely to be major contributors to PKDL susceptibility. Given the growing interest in the role that rare variants in orphan diseases can play in translation of genetic research [28], our study makes an important general contribution to knowledge of population variants of potential relevance to Sudan.

The present study also highlighted a cluster of upstream CNSs likely to contain the regulatory elements influencing *IFNGR1* expression. In particular, carriage of alternative alleles at rs55995532, rs1327473, and rs17175050, rs56178591, rs17175057 result in loss or gain of transcription factors C/EBPbeta, AP-1, NF-1/GATA-1/ ZBTB3, MAZ/ ZNF263, and SP1/ KLF7, respectively. Again these polymorphisms are unlikely to be of direct importance to PKDL in this community, although genotyping is required to establish their frequency in this population. Certainly there is potential for functional importance of these SNPs in relation to immune responses. For example, the CEBPbeta transcription factor is important in the regulation of genes involved in immune and inflammatory responses and has been shown to bind to the interleukin (IL)-1 response element in the IL-6 gene, as well as to regulatory regions of several acute-phase and cytokine genes [29]-[32]. Therefore, it will be important to test whether these polymorphisms are associated with PKDL and/or in linkage disequilibrium with the *IFNGR1* rs141836145 (−270 T/C) SNP or other variants shown to be on risk versus protective haplotypes previously found to be associated with PKDL [7].

IFNG mediates host protection against leishmaniasis, exerting its pleotropic effect in macrophage activation and killing *Leishmania* parasites through induction of nitric oxide [33]. A previous report on mRNA transcripts in a single patient with PKDL documented strong expression of both Th1 and Th2 cytokines and the absence or undetectable expression of these cytokines in normal skin biopsy samples [34]. In the present study the level of IFNG expression in PKDL patients was undetectable, as it was in the control commercially acquired skin sample. This is in contrast to PKDL in India where elevated expression of intra-lesional IFNG compared to control skin tissue was previously observed [35]. Elevated levels of intra-lesional IFNG mRNA have also been demonstrated in non-healing patients with CL compared to early lesions [36]. These results suggest that, in contrast to Indian PKDL and non-healing CL, the failure of macrophages to become activated during Sudanese PKDL may be due to the absence of an intra-lesional Th1-type response. Of interest we found that mRNA for IFNGR1, although detectable in some patients, was also uniformly low in all Sudanese PKDL biopsies relative to the endogenous housekeeper gene beta-actin. Ansari and colleagues [35] also observed minimal intra-lesional IFNGR1 expression in Indian PKDL [35]. This suggests that low or variable expression of IFNGR1, rather than presence or absence of IFNG *per se*, could be the common feature of PKDL across the geographical and clinical phenotypic divides of India and Sudan. It is possible that this low IFNGR1 expression level may lead to a gap in downstream signaling. Almost all cell types express IFNGR1, but it is of particular importance to the macrophage, which requires IFNG for control of intracellular parasites, such as *Leishmania*. In our previous studies, PKDL development was shown to be associated with *IFNGR1* polymorphisms [6],[7]. The detection here of a number of regulatory elements within the novel polymorphisms and CNSs may influence *IFNGR1* expression.

PKDL is an unusual dermatosis that develops in more than 50% of apparently cured VL cases in Sudan [37],[38]. So far, little is known about the parasite/host factors that drive the parasite to shift from the site of infection to the dermis. Inadequate treatment is considered to be a factor in PKDL development. However the disease may develop even after complete treatment. To study whether PKDL is influenced by low expression of IFNGR1, changes in IFNG and IFNGR1 levels upon treatment were analysed in 24 VL patients. Our results showed that, whilst highly variable between individuals, the level of IFNG expression in lymph nodes aspirates from VL patients was significantly higher in paired pre-treatment compared to post-treatment samples. The results are in agreement with observations regarding increased levels of mRNA encoding IFNG in bone marrow aspirates and lymph nodes from African patients compared to endemic and U.S. control groups [39]. High levels of IFNG are also observed in *Leishmania* antigen-stimulated whole blood from VL patients in India [40],[41], although this response is lost in preparation of PBMCs from the same patients consistent with low levels of IFNG observed in stimulated PBMCs from studies of other populations [42],[43]. This loss of IFNG response between whole blood and PBMC may be due to loss of some factor (which has a positive effect on IFNG production in active VL) during the preparation of PBMC, and/or to the loss of a specific CD4 T cell population during PBMC preparation [40]. We and others [34],[39],[40],[44] suggest that, during active visceral leishmaniasis, the immune system is not suppressed but is highly activated.

The alpha chain encoded by the *IFNGR1* gene is the IFNG binding subunit while the signal transduction subunit is the beta chain. The importance of the alpha chain for IFNG responsiveness has already been studied. For example, it has been shown that intra-dermal injection of even very low numbers of infective *L. major* promastigotes in mice knocked-out for the *Ifngr1* gene on a genetically resistant mouse background induced disseminated fatal disease despite mounting a Th1 response in these mice [45]. In this study, we found that the expression level of *IFNGR1* mRNA in active VL was highly variable between individuals, and unchanged pre- and post-treatment in paired samples. This variability may relate to observations made by other groups. Kariminia and coworkers [46] showed that *Leishmania* infection induces down-regulation of *IFNGR1* on CD45+ cells of the draining lymph nodes. Another report has demonstrated down regulation of *IFNGR1* in PBMC from VL cases as well as in the THP1 monocytic cell line infected with *Leishmania* [47]. Low expression of IFNGR1 in some VL cases could provide an indicator of those individuals likely to progress to PKDL following drug-cure.

## Conclusions

Most important amongst our findings was the uniformly low expression of IFNGR1 in PKDL skin biopsies from Sudanese patients, which now provides commonality with Indian PKDL. This could explain parasite persistence and is consistent with prior demonstration of genetic association of PKDL with *IFNGR1* promoter polymorphisms. Further work is required to determine whether the additional putative functional variants in highly conserved regions in the promoter and upstream regions identified here contribute to this association. The identification of novel potentially functional rare variants in the *IFNGR1* promoter in this Sudanese population, whilst unlikely to contribute directly to risk and protective haplotypes for PKDL, make an important general contribution to knowledge of rare variants of potential relevance in this Sudanese population.

## Authors’ original submitted files for images

Below are the links to the authors’ original submitted files for images. Authors’ original file for figure 1 Authors’ original file for figure 2
