# Supplementary figures and images for: Insights into the possible role of IFNG and IFNGR1 in Kala-azar and Post Kala-azar Dermal Leishmaniasis in Sudanese patients
Source: BMC Infect Dis. 2014 Dec 3;14:662. doi: 10.1186/s12879-014-0662-5 (PMC4265480; doi:10.1186/s12879-014-0662-5)

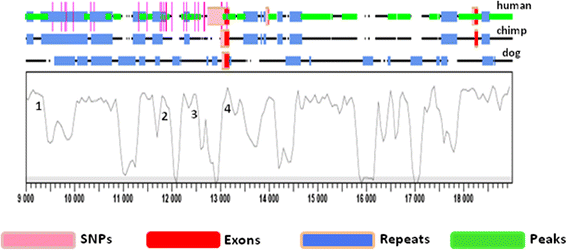

Supplement: Supplementary file 1 — Authors’ original file for figure 1 [file 12879_2014_662_MOESM1_ESM.gif]

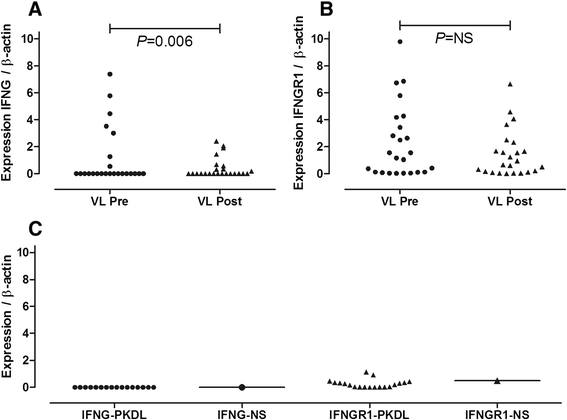

Supplement: Supplementary file 2 — Authors’ original file for figure 2 [file 12879_2014_662_MOESM2_ESM.gif]
